# FGFR1OP tagSNP but Not CCR6 Polymorphisms Are Associated with Vogt-Koyanagi-Harada Syndrome in Chinese Han

**DOI:** 10.1371/journal.pone.0069358

**Published:** 2013-07-23

**Authors:** Xianglong Yi, Liping Du, Shengping Hou, Fuzhen Li, Yuanyuan Chen, Aize Kijlstra, Peizeng Yang

**Affiliations:** 1 The First Affiliated Hospital of Chongqing Medical University, Chongqing Key Laboratory of Ophthalmology, Chongqing Eye Institute, Chongqing, China; 2 Department of Ophthalmology, The First Affiliated Hospital, Xinjiang Medical University, Urumqi, Xinjiang, China; 3 University Eye Clinic Maastricht, Maastricht, The Netherlands; Keio University School of Medicine, Japan

## Abstract

**Background:**

Polymorphisms of the CC chemokine receptor 6 (CCR6) and FGFR10P tagSNP (locus close to CCR6) at 6q27 have recently been reported to be associated with the susceptibility to several immune-related diseases. This study was designed to determine the association of CCR6 and FGFR10P (tag)SNPs with Vogt-Koyanagi-Harada (VKH) syndrome, an autoimmune disease directed against melanocytes, in two independent Chinese Han populations.

**Methodology/Principal Findings:**

A total of 601 VKH patients and 725 healthy controls from two Chinese Han populations were genotyped by the polymerase chain reaction-restriction fragment length polymorphism method. Hardy-Weinberg equilibrium was tested using the χ^2^ test. Genotype frequencies were estimated by direct counting. Allele and genotype frequencies were compared between patients and controls using the χ^2^ test. The frequency of the A allele of rs2301436 was significantly higher both in Cohort 1 and Cohort 2 as compared with two separate controls (P = 0.044; P = 0.049, respectively). The significance was lost after Bonferroni correction in both cohorts (Pc = 0.516; Pc = 0.392, respectively). The frequency of the A allele was significantly higher in the combined patient group as compared with all controls before and after Bonferroni correction (P = 0.005, Pc = 0.025). The genotype and allele frequencies of rs3093024, rs6902119, rs3093023 and rs968334 were not different between patients with VKH and healthy controls based on analysis either for both cohorts or for the patients and controls in total. Analysis according to extra ocular clinical findings including headache, alopecia and poliosis, vitiligo and tinnitus did not show any association of the five polymorphisms with these parameters.

**Conclusion:**

These results suggest that the rs2301436 tagSNP of FGFR10P is positively associated with susceptibility to VKH syndrome in the tested Chinese Han populations. No association was found for the tested CCR6 SNPs.

## Introduction

Vogt-Koyanagi-Harada (VKH) syndrome is an autoimmune disease characterized by severe bilateral panuveitis frequently associated with poliosis, vitiligo, alopecia and central nervous system and auditory signs [1], [2], [3]. Although the etiology of VKH syndrome remains unclear, studies have revealed that an autoimmune response directed against melanocytes plays a key role in the initiation and maintenance of this disease [4], [5]. However, the factors which trigger the autoimmune response are still unknown. Like most complex traits diseases, a widely accepted hypothesis is that viral infection or cutaneous injury triggers an inappropriate and overactive T cell-mediated autoimmune response [6], [7], [8], which in turn leads to tissue damage in multiple organs in genetically susceptible individuals. The genetic background is still not completely understood although a number of HLA and non-HLA genes have been shown to be associated with this disease [9], [10], [11], [12]. Therefore, further studies on the association of relevant genes with VKH syndrome may provide further evidence for the genetic background of this disease.

An LD block on chromosome 6 in the region of the genes encoding for CCR6 and FGFR1OP (6q27) has been shown to be associated with an increased risk for a variety of autoimmune diseases, such as rheumatoid arthritis (RA), Crohn’s disease and vitiligo [13], [14], [15], [16]. These findings suggest that this region may contain a common risk gene for a number of autoimmune disorders although the exact gene has not yet been identified. Of interest is the fact that vitiligo is also a clinical feature of VKH. Whether genes in this region are also associated with VKH is not yet known and was therefore the subject of this study.

Our results suggest that the rs2301436 tagSNP of FGFR10P is positively associated with the susceptibility to VKH syndrome in the tested Chinese Han populations, whereas no associations were found for the SNPs tested in the region encoding CCR6.

## Results

There was no difference in age and gender distribution between VKH patients and controls in two independent Chinese case-control cohorts (Table 1). Clinical findings of VKH patients are presented in Table 2. All samples from 601 VKH patients and 725 controls were genotyped for five SNPs including rs3093024, rs6902119, rs3093023 and rs968334 in the CCR6 and the rs2301436 FGFR10P tagSNP. The genotype and allele frequencies of the five tested SNPs examined in VKH patients and normal controls are summarized in Table 3. The distribution of genotype frequencies of each SNP in the two cohorts were in Hardy–Weinberg equilibrium. The frequency of the A allele of rs2301436 was significantly higher both in Cohort 1 and Cohort 2 as compared with two separate controls (P = 0.044; P = 0.049, respectively). The significance was lost after Bonferroni correction (Pc = 0.516; Pc = 0.392, respectively). However, based on the analysis for the combined patient and control groups, a significantly higher frequency of the A allele of rs2301436 was observed in the VKH patients (P = 0.005). This difference remained significant after Bonferroni correction (Pc = 0.025, odds ratio = 1.25, 95% CI 1.07 to 1.45). The same result was observed in the G allele of rs2301436 (Pc = 0.025, odds ratio = 0.80, 95% CI 0.69 to 0.94). A significantly lower frequency of the rs2301436 GG genotype was also observed in cohort 2 (P = 0.043). This difference was lost after Bonferroni correction (Pc = 0.516). Taking the two cohorts together, there was also no significant difference between patients and controls after Bonferroni correction (P = 0.006, Pc = 0.072). There were no differences in the allele or genotype frequencies of rs3093024, rs6902119, rs3093023 and rs968334 SNPs between VKH patients and controls. We also performed a study on the influence of gender and clinical parameters, including headache, alopecia and poliosis, vitiligo and tinnitus on the association of the tested five SNPs with VKH syndrome. The results showed that there was no detectable association of these parameters with these SNPs. Linkage disequilibrium(ID) between four SNPs including rs3093024, rs6902119, rs3093023 and rs968334 is strong, however we did not find a tight LD between rs2301436 and the other four SNPs in the CCR6 gene after analysis of LD(data not shown).

**Table 1:** Age and gender distribution in VKH patients and controls.

	Cohort 1		Cohort 2		Total	
	VKH patients(n = 318)	Controls(n = 346)	VKH patients(n = 283)	Controls(n = 379)	VKH patients(n = 601)	Controls(n = 725)
Age (y) (mean±SD)	34.8±7.3	36.7±5.9	34.7±7.2	37.2±6.2	34.5±9.7	36.7±10.9
Male	177(51.5)	193(55.8)	151(52.8)	191(50.4)	328 (51.7)	384 (52.9)
Female	141(48.5)	153(44.2)	132(47.2)	188(49.6)	273 (49.3)	341 (47.1)

**Table 2:** Clinical characteristic of VKH patients.

Extraocular findings	Cohort 1		Cohort 2		Total	%
VKH patients (n = 601)	318		283		601	
Headache	167	52.5	146	51.5	313	52.1
Alopecia and poliosis	153	48.1	134	47.3	287	47.8
Vitiligo	76	23.9	66	23.3	142	23.6
Tinnitus	152	47.8	122	43.1	277	46.1

**Table 3:** Frequencies of alleles and genotypes of CCR6 and FGFR10P polymorphisms in VKH patients and controls.

SNP	Genotype allele			VKH				Controls							P Value								OR(95%CI)						
					Cohort1	Cohort2	Total			Cohort1		Cohort2		Total		Cohort1		Cohort2		Total P/Pc		Cohort1			Cohort2	Total			
rs3093024			GG		107(33.9)	109(38.5)	216(35.9)			128(37.0)		144(37.9)		272(37.5)		0.384		0.939		0.530/NS		0.868(0.631–1.194)			1.013(0.734–1.397)	0.930(0.742–1.166)			
			AA		60(18.9)	30(10.6)	90(15.0)			50(14.4)		53(13.9)		103(14.2)		0.706		0.227		0.618/NS		1.089(0.698–1.701)			0.743(0.456–1.204)	1.081(0.795–1.470)			
			AG		151(47.2)	144(50.9)	295(49.1)			168(48.6)		182(48.2)		350(48.3)		0.750		0.194		0.553/NS		0.952(0.701–1.291)			1.256(0.890–1.773)	1.076(0.844–1.371)			
			A		271(42.6)	204(36.0)	475(39.5)			268(38.7)		288(38.0)		556(38.3)		0.927		0.521		0.856/NS		1.011(0.791–1.293)			0.928(0.738–1.166)	0.984(0.826–1.172)			
			G		365(57.4)	362(64.0)	727(60.5)			424(61.3)		470(62.0)		894(61.7)		0.927		0.521		0.856/NS		0.989(0.773–1.264)			1.078(0.857–1.355)	1.016(0.826–1.172)			
rs6902119			CC		115(36.3)	115(40.8)	230(38.4)			135(39.0)		166(43.8)		301(41.5)		0.467		0.437		0.249/NS		0.890(0.650–1.219)			0.884(0.647–1.208)	0.878(0.704–1.095)			
			TT		45(14.2)	41(14.5)	86(14.4)			45(13.0)		54(14.2)		99(13.7)		0.188		0.916		0.714/NS		0.739(0.470–1.161)			1.024(0.660–1.588)	1.060(0.776–1.448)			
			CT		158(49.5)	127(44.7)	285(47.2)			166(48.0)		159(42.0)		325(44.8)		0.690		0.484		0.380/NS		1.064(0.784–1.443)			1.118(0.819–1.525)	1.102(0.887–1.370)			
			T		247(38.9)	208(36.9)	455(38.0)			256(37.0)		267(35.2)		523(36.1)		0.461		0.535		0.311/NS		1.087(0.871–1.357)			1.074(0.856–1.348)	1.085(0.926–1.272)			
			C		387(61.1)	356(63.1)	743(62.0)			436(63.0)		491(64.8)		927(63.9)		0.461		0.535		0.311/NS		0.920(0.737–1.149)			0.931(0.742–1.168)	0.921(0.786–1.179)			
rs3093023			AA		81(25.6)	54(19.1)	135(22.6)			82(23.7)		76(20.1)		158(21.9)		0.580		0.772		0.745/NS		1.105(0.776–1.574)			0.944(0.640–1.393)	1.044(0.805–1.355)			
			GG		85(26.8)	104(36.9)	189(31.5)			98(28.3)		120(31.6)		218(29.9)		0.664		0.161		0.560/NS		0.927(0.659–1.304)			1.261(0.912–1.745)	1.072(0.848–1.355)			
			AG		152(47.6)	125(44.0)	277(45.9)			166(48.0)		183(48.3)		349(48.2)		0.930		0.168		0.419/NS		0.986(0.727–1.338)			0.785(0.556–1.108)	0.914(0.736–1.136)			
			G		321(50.6)	332(58.9)	653(54.5)			330(47.7)		335(44.2)		665(45.9)		0.541		0.266		0.849/NS		0.935(0.754–1.160)			1.133(0.909–1.413)	1.105(0.870–1.184)			
			A		313(49.4)	232(41.1)	545(45.5)			362(52.3)		423(55.8)		785(54.1)		0.541		0.266		0.849/NS		1.070(0.862–1.327)			0.882(0.708–1.100)	0.985(0.845–1.149)			
rs968334			GG		135(35.6)	115(33.2)	250(34.5)		93(32.9)		99(31.1)		192(31.9)		0.460		0.562		0.329/NS		0.515(0.505–0.525)			0.621(0.612–0.631)			0.342(0.333–0.351)		
			AA		55(14.5)	54(15.6)	109(15.0)			36(12.7)		62(19.5)		98(16.3)		0.508		0.187		0.525/NS		0.572(0.562–0.581)			0.217(0.209–0.225)	0.543(0.533–0.552)			
			AG		189(49.9)	177(51.2)	366(50.5)			154(54.4)		157(49.4)		311(51.7)		0.247		0.646		0.647/NS		0.271(0.262–0.280)			0.700(0.691–0.709)	0.657(0.647–0.666)			
			A		299(39.4)	285(41.2)	584(40.3)			226(39.9)		281(44.2)		507(42.2)		0.859		0.270		0.321/NS		0.862(0.856–0.869)			0.295(0.286–0.304)	0.322(0.313–0.331)			
			G		459(60.6)	407(58.8)	866(59.7)			340(60.1)		355(55.8)		695(57.8)		0.859		0.270		0.321/NS		0.868(0.861–0.875)			0.290(0.281–0.299)	0.326(0.317–0.335)			
rs2301436		GG		56(17.6)		54(19.1)	110(18.3)			81(23.4)		98(25.6)		179(24.7)		0.068		0.043		0.006/NS		0.702(0.480–1.028)			0.679(0.467–0.988)	0.686(0.525–0.896)			
		AA		101(31.8)		93(32.8)	194(32.3)			92(26.6)		109(28.8)		201(27.7)		0.136		0.244		0.065/NS		1.291(0.932–1.806)			1.219(0.873–1.701)	1.249(0.986–1.581)			
		AG		161(50.6)		136(48.1)	297(49.4)			173(50.0)		172(45.3)		345(47.6)		0.903		0.526		0.547/NS		1.019(0.751–1.577)			1.105(0.811–1.505)	1.069(0.861–1.327)			
		A		363(57.1)		322(56.9)	685(57.0)			357(51.6)		390(51.5)		747(51.5)		0.044		0.049		**0.005/0.025**		1.249(1.006–1.551)			1.246(1.001–1.552)	1.248(1.070–1.456)			
		G		273(42.9)		244(43.1)	517(43.0)			335(48.4)		368(48.5)		703(48.5)		0.044		0.049		**0.005/0.025**		0.801(0.645–0.994)			0.802(0.644–0.999)	0.801(0.687–0.935)			

OR = odds ratio; 95%CI = 95% confidence interval; Pc = P values were corrected with the Bonferroni correction; NS = not significant.

## Discussion

In this study, we investigated whether polymorphisms of CCR6 including rs3093024, rs6902119, rs3093023, rs968334 and the FGFR10P tagSNP rs2301436 contributed to development VKH in two independent Chinese case-control cohorts. Our results showed that the A allele of rs2301436 was associated with susceptibility to VKH syndrome.

As the CCR6 gene and loci close to CCR6 have been proven to be associated with a number of autoimmune diseases, we examined whether there was an association of this gene with VKH syndrome, a well known autoimmune disease involving the eye, the neurologic/auditory and integument organs. The epidemiology of VKH syndrome in China is not available and therefore data on its exact prevalence are unknown. According to our studies published in 2005 [17], VKH syndrome is one of the most common uveitis entities in China. All the patients recruited in our study were diagnosed according to the criteria suggested by the First International Workshop criteria for VKH syndrome [18]. In our clinical practice, VKH syndrome in Chinese patients is characterized by early posterior uveitis and, if not properly controlled, spreads to the front of the eye leading to an anterior uveitis simulating a nongranulomatous inflammation, which is followed by a subsequent granulomatous panuveitis [19]. To date no controlled studies have been performed to compare the clinical features of VKH syndrome among various ethnic populations. Whether genetic susceptibility is different among VKH patients around the globe also deserves further study.

The selection of SNPs around CCR6 were based on earlier autoimmune disease associations reported by others, including a number of autoimmune diseases, including RA, Crohn’s disease and vitiligo [13], [14], [15], [16]. Autoimmunity against melanocytes is an important feature in both VKH and vitiligo and therefore prompted us to investigate the four CCR6 SNPs and one FGFR10P tagSNP as candidates in our study. The association of these five SNPs with VKH was investigated in two independent Chinese case-control cohorts. A number of efforts were made to ensure an accurate result. First, the patients were diagnosed strictly according to the First International Workshop criteria for VKH syndrome. Patients with a doubtful diagnosis were excluded. Second, the controls were age- and ethnicity-matched with patients and all patients were chosen from a Chinese Han population to avoid the influence of ethnic variants on the results. Third, direct sequencing was performed on 10% of the tested samples and the results were identical to those of genotyping by PCR-restriction fragment length polymorphism.

Our results showed no association of 4 SNPs out of the 5 tested SNPs, i.e. rs3093024, rs6902119, rs3093023 and rs968334 in the CCR6 gene, with VKH syndrome. Initially we also did not find an with the FGFR10P SNP rs2301436 according to a separate analysis of two independent Chinese case-control cohorts. However, a significant association was observed when considering the two cohorts as a whole. This result seems to suggest that a sufficient number of the tested patients is necessary in studying the association of candidate genes with a certain disease. The association of the FGFR10P tagSNP rs2301436 with VKH syndrome was similar to that reported previously in Crohn’s disease [14], in which the A allele of rs2301436 was considered as a protective allele to this disease. It is not clear whether the A allele of rs2301436 contributes to the predisposition to VKH syndrome by itself, or is just a marker in linkage disequilibrium with the true susceptibility gene. The absence of an association of the other four SNPs with VKH presented here is not consistent with the results reported in other autoimmune diseases including RA and vitiligo in European and Asian populations [13], [14], [15]. This might be explained by the fact that the etiology and pathogenesis of VKH is different compared to the other autoimmune diseases [17], [19]. The other possibility could be that Chinese Han people have a different genetic background as compared to other ethnic populations.

It is worthwhile to point out that several limitations exist in our study. Although the patients enrolled in this study were from two independent Chinese case-control cohorts, only Chinese Han individuals were included. Therefore, the results presented here need to be confirmed using different ethnic populations. As mentioned above, there are no epidemiologic data on the prevalence of VKH syndrome available in China, and it is therefore not clear whether the population size is sufficient to detect an effect according to a power calculation. Although the association with the FGFR10P gene suggests that these polymorphisms may affect T cell mediated responses we did not perform biological function tests in control carriers of the various phenotypes.

In conclusion, this study indicates that the A allele of the FGFR10P tagSNP rs2301436, but not rs3093024, rs6902119, rs3093023 and rs968334 in the CCR6 gene, is associated with susceptibility to VKH syndrome in Chinese Han.

## Subjects and Methods

### Study Population

To investigate the association with CCR6 and FGFR10P, we performed a replication study using two independent Chinese case-control cohorts. Cohort 1 consisted of 318 cases that were referred to Zhongshan Ophthalmic Center, Sun Yat-sen University from January 2005 to March 2008 and 346 controls during the same period. Cohort 2 consisted of 283 cases that were referred to the First Affiliated Hospital of Chongqing Medical University from April 2008 to January 2011 and 379 controls at the same period (Table 1). The clinical characteristics of VKH patients were assessed at the time of diagnosis and are summarized in Table 2. All control subjects were matched ethnically and geographically with the patients. The diagnosis of VKH was made according to the First International Workshop criteria for VKH syndrome [18]. This study was approved by the Ethics Committee of Zhongshan Ophthalmic Center and the First Affiliated Hospital of Chongqing Medical University, and written informed consent was obtained from all the subjects. All procedures followed the tenets of the Declaration of Helsinki.

### Genomic DNA Extraction and Genotyping

**Figure 1:**
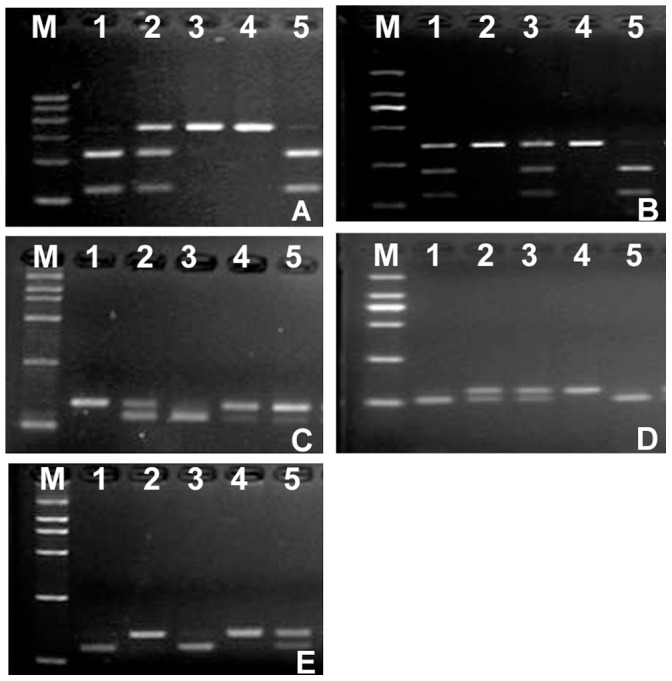
Example of the differrent genotype of five SNPs. (A) rs3093024: laneM, molecular size standard; lane 1,5, genotype GG(217 bp and 123 bp); lane 2, genotype AG(340 bp, 217 bp and 123 bp); and lane 3,4, genoty (340 bp). (B) rs6902119: lane 1,3, genotype CT(366 bp, 228 bp and 138 bp); lane 2,4, genotype CC(366 bp); and lane 5, genotype TT(228 bp and 138 bp). (C) rs3093023: lane 1, genotype AA(114 bp); lane 2,4, 5, genotype AG(114 bp and 27 bp); and lane 3, genotype GG(27 bp). (D) rs968334: lane 1,5, genotype GG(20 bp); lane 2,3, genotype AG(100 bp and 20 bp); and lane 4, genotypeAA(100 bp). (E) rs2301436: lane 1,3, genotype GG(21 bp); lane 2,4, genotype AA(120 bp); and lane 5, genotype AG(120 bp and 21 bp).

Blood samples were collected in ethylenediaminetetraacetate tubes and kept at −70°C until used. Genomic DNA was isolated from blood leukocytes by using the commercial kit QIAamp DNA Blood Mini kit (Qiagen, Valencia, CA). The extracted DNA was stored at 20°C until used. Amplification of the target DNA in the CCR6 gene was analyzed by the polymerase chain reaction (PCR) using primers presented in Table 3. Each PCR reaction was carried out in a 10 µl reaction mixture containing 5 µl Premix Taq (Ex Taq Version; TaKaRa Biotechnology, Co, Ltd., Dalian, China), 20 pmol primers, and 0.2 µg of genomic DNA for amplification of the DNA. Its conditions were as follows: initial denaturation at 95°C for 5 minutes followed by 40 cycles of denaturation at 94°C for 30 seconds, annealing at different temperatures (51°C for rs3093024, 55°C for rs6902119, 60°C for rs3093023, and 57°C for rs968334 and rs2301436) for 30 seconds, extension at 72°C for 30 seconds, and a final extension at 72°C for 5 minutes. These SNPs were genotyped by PCR-restriction fragment length polymorphism analysis. PCR products of rs3093024, rs6902119, rs3093023, rs968334 and rs2301436 polymorphisms were respectively digested with 2 U of BtsI (New England Biolabs, Inc., Ontario, Canada), PvuII (New England Biolabs, Inc., Ontario, Canada), TspRI (New England Biolabs, Inc., Ontario, Canada), and MspI (New England Biolabs, Inc., Ontario, Canada) restriction enzymes (Table 4) in a 10 µl reaction volume overnight. Digestion products were visualized on a 3.5% agarose gel and stained with GoldView (SBS Genetech, Beijing, China)(Figure 1). Direct sequencing was also preformed in Invitrogen Biotechnology Company, using the randomly selected subjects (10% of all samples) to validate the method used in this study.

**Table 4:** Primers of CCR6 SNPs and FGFR10P restriction enzymes used for PELP analysis.

Rs number	Primers	Restriction enzyme
rs3093024	5′-CCCACCTGTCCTTACTGC-3′	BtsI
	5′-CATTTACTTCCCACCTTTT-3′	
rs6902119	5′-GGGAATGACTGGCTATGA-3′	PvuII
	5′-TTCTGCGTGAACATGAATT-3′	
rs3093023	5′-CCTCACAGTGTCTATGCAAATGAACA-3′	TspRI
	5′-GTCACCCTGAGAAAGCTGAGACCT-3′	
rs968334	5′- GAGGGGCTGGCTTTGTGCCG -3′	MspI
	5′- CCATCCCACCTTCGTCAGCACG -3′	
rs2301436	5′-TTGACCTCTTCACTGTGATTTTTTCC-3′	MspI
	5′-ATCAGCATGGCTGTTAGTGGCT-3′	

### Statistical Analysis

Hardy-Weinberg equilibrium was tested using the χ^2^ test. Genotyping results of the manuscript Genotype frequencies were estimated by direct counting. Allele and genotype frequencies were compared between patients and controls by the χ^2^ test using SPSS for Windows (version 13.0; SPSS, Inc., Chicago, IL). The association of the tested SNPs with the extraocular findings was evaluated by logistic regression. All statistical tests were two-sided, The P-values were corrected (Pc) with the Bonferroni correction by multiplying with the number of analyses performed. The Pc <0.05 was considered as significant.
